# Enhanced Production of ECM Proteins for Pharmaceutical Applications Using Mammalian Cells and Sodium Heparin Supplementation

**DOI:** 10.3390/pharmaceutics14102138

**Published:** 2022-10-08

**Authors:** Javier Garcia-Pardo, Sergi Montané, Francesc Xavier Avilés, Sebastian Tanco, Julia Lorenzo

**Affiliations:** Institut de Biotecnologia i Biomedicina and Departament de Bioquímica i Biologia Molecular, Universitat Autònoma de Barcelona, 08193 Bellaterra, Barcelona, Spain

**Keywords:** ECM proteins, sodium heparin, heparin binding, enhanced protein expression, carboxypeptidases, mammalian cells, HEK 293F cells, carboxypeptidase A6, carboxypeptidase Z, thrombin-activable fibrinolysis inhibitor

## Abstract

The yields of soluble ECM proteins recombinantly produced with mammalian cells can be significantly enhanced by exploiting the stabilizing properties of heparin. Here, we propose a simple and straightforward scalable protocol for the mammalian cell production of ECM proteins with affinity for heparin, using heparin as a supplement. As proof of concept, we have demonstrated the high-level expression of four biomedically relevant human enzymes such as carboxypeptidase Z (CPZ), carboxypeptidase A6 (CPA6), beta-galactoside alpha-2,6-sialyltransferase 2 (ST6GAL1) and thrombin-activable fibrinolysis inhibitor (TAFI). We found a strong linear correlation between the isoelectric point (pI) of a protein and the improvement in protein expression levels upon heparin addition, providing a reference for selecting novel protein targets that would benefit from heparin supplementation. Finally, we demonstrated the compatibility of this approach with a three-step purification strategy that includes an initial heparin affinity purification step. Using CPZ as a representative example, we performed a preparative purification of this enzyme. The purified protein is enzymatically active and can be used for pharmaceutical applications as well as for high-throughput functional and structural studies.

## 1. Introduction

The rapid progress of the biopharmaceutical industry has created a constant need to produce a wide variety of structurally complex recombinant proteins [1,2,3,4]. To achieve the production of functional proteins it is often required to incorporate post-translational modifications, molecular chaperones and/or co-factors to support their elaborated folding and enzymatic activity [1,5]. Thus, the use of mammalian expression platforms has been increasing because they are able to produce complex eukaryotic proteins that are otherwise problematic to express in other systems [2,3,5]. In particular, the utilization of suspension-grown mammalian host cell lines provides a highly homogeneous and scalable platform to produce pharmaceutical proteins under native-like conditions [3,6]. Examples of recently approved pharmaceutical products obtained with this system include monoclonal antibodies, hormones, clotting factors, and enzymes [1,7]. Moreover, mammalian cells are of great importance for research purposes, to produce structural complex recombinant proteins in structural biology at a laboratory scale [6]. However, despite recent advances in the field, this expression system still suffers from significant limitations, especially with “difficult-to-express” proteins [8,9]. This is the case of several secreted proteins that bind to the extracellular matrix (ECM) [10,11].

The ECM is a multifunctional scaffolding structure that provides structural and biochemical support to cells and their secreted protein factors. The ECM is built up of numerous macromolecules such as collagens, elastin, microfibrillar proteins, proteoglycans (e.g., hyaluronan) and non-collagenous glycoproteins [11,12]. In addition to these structural components, many secreted molecules can strongly bind to the ECM scaffolding proteins due to their heparin-binding properties [13]. Among ECM-binding proteins, several types of growth factors, proteases and relevant signaling molecules have been described so far [11,14]. The affinity of these proteins to the ECM is often mediated by their specific binding to heparan sulfate proteoglycans and other heparin-like molecules [15,16]. Through this binding, the ECM protects such proteins from proteolytical degradation [17] and can fine-tune their biological responses [18]. Not surprisingly, ECM and ECM-associated factors are involved in human diseases, since they mediate essential biological processes such as cell adhesion, cell communication and differentiation [19,20]. Therefore, recent therapeutic strategies have focused on utilizing ECM molecules for the development of novel therapeutics [21]. However, the production of ECM-binding proteins as soluble molecules is often still a difficult task due to their inherent capacity for ECM binding and aggregation.

Here we describe a simple and highly efficient method for expressing ECM-binding proteins in suspension-grown mammalian cells. This approach combines transient expression in Human Embryonic Kidney 293F (HEK 293F) cells with the addition of sodium heparin two days after transfection to improve the protein stability of ECM-bound proteins. To test the robustness of this pipeline, the expression of seven different biomedically relevant secreted enzymes was evaluated. A robust enhanced expression of all ECM-binding proteins was observed, demonstrating that the addition of heparin enables the soluble accumulation, and thus higher production yields, of those ECM/heparin-binding molecules. We also show that this approach allows protein purification of soluble and active enzymes such as carboxypeptidase Z (CPZ), an ECM-bound enzyme involved in C-terminal neuropeptide processing. All in all, we report a cost-effective protein expression system that boosts the recombinant production of ECM-binding proteins suitable for pharmaceutical applications.

## 2. Materials and Methods

### 2.1. Plasmids and Cell Culture

Plasmids for mammalian expression of carboxypeptidase D (CPD, UniProtKB accession: O75976), carboxypeptidase Z (CPZ, UniProtKB accession: Q66K79), carboxypeptidase O (CPO, UniProtKB accession: Q8IVL8), thrombin-activable fibrinolysis inhibitor (TAFI, UniProtKB accession: Q96IY4) and beta-galactoside alpha-2,6-sialyltransferase 1 (ST6GAL1, UniProtKB accession: P15907) were obtained by subcloning the respective genes into the pTriEx-7 (Merck Millipore, Burlington, MA, USA) expression vector. All these constructs express the protein of interest fused to an N-terminal IgM signal sequence plus an N-terminal Strep-tag® (Trp-Ser-His-Pro-Gln-Phe-Glu-Lys), as previously described [22,23,24]. The plasmid pcDNA3.1(-)hCPA6-HA-H6 was generously provided by Prof. Peter Lyons (Andrews University, MI, USA) and was constructed by subcloning human pro-CPA6 gene (UniProtKB accession: Q8N4T0) into the EcoRI/BamHI sites of pcDNA3.1(-) [13]. The pOPINF-α-gal expression vector was kindly provided by Dr. J. L. Corchero (Universitat Autònoma de Barcelona, Spain). In this vector, α-gal (UniProtKB accession: P06280) production is under the control of CMV enhancer and chicken b-actin (CBA) promoter and includes an additional 6xHis tag at the protein C-terminus [25].

FreeStyle™ 293F cells (HEK 293F cells, Thermo Fisher Scientific, Waltham, MA, USA) were grown in FreeStyle 293 expression medium (Thermo-Fischer Scientific) in flasks on a rotary shaker (120 rpm) at 37 °C and in a humidified atmosphere with 8% CO_2_. For maintenance, the cell culture was diluted each 48–72 h, to maintain the cells at a density between 0.2 × 10^6^ and 3.0 × 10^6^ cells/mL. Since the FreeStyle 293 expression medium is a ready-to-use medium, no additional supplementation is required.

### 2.2. Step-by-Step Protein Expression Procedure

E.1 Dilute HEK 293F cells at a density of 0.5 × 10^6^ cells/mL into a final volume of 450 mL into a 2-L shaker flask.

Note 1: This protocol is optimized for a large-scale expression of heparin-binding proteins but is suitable for any scale of expression with slight modifications. To adapt this protocol, all the volumes and quantities of reagents should be scaled proportionally.

Note 2: It is widely accepted that cell culture flasks can accommodate a minimum of 1/10 of their nominal volume to a maximum of 1/4 of their nominal volume of suspension culture. For larger expression volumes, we recommend using multiple culture flasks.

E.2. Incubate the cells for 24 h in an orbital shaker incubator at 37 °C, 120 rpm and in a humidified atmosphere with 8% CO_2_ until cells reach a density of 1.0 × 10^6^ cells/mL.

E.3. Pipette a total of 500 µg of DNA into 50 mL of FreeStyle 293 expression medium and vortex the sample for 30 s (1 µg of DNA per mL of final cell culture).

Note 3: Here we used three different mammalian expression vectors such as pTriEx-7, pcDNA3.1 or pOPINF. However, any other vector that allows for an efficient mammalian protein expression to the extracellular medium can be used for this protocol. It is advisable to use plasmid DNA of the highest quality/purity and suitable for cell culture. Typically, DNA should be sterile and endotoxin-free, as well as free from other typical DNA extraction contaminants.

E.4. Add 1.5 mL of a filter-sterilized, 1.0 mg/mL polyethylenimine (PEI linear, 25,000 Da, Polysciences, Warrington, PA, USA) in water solution to the DNA mix and vortex vigorously for 30 s. The final DNA–PEI ratio in the transfection mix is 1:3 (*w*/*w*).

E.5. Incubate the mixture at room temperature for 15–20 min to enable DNA/PEI complex formation.

E.6. Gently, add the DNA/PEI transfection mix to the cells.

Note 4: Under optimal conditions, cells are transfected when they reach a density of about 1.0 × 10^6^ cells/mL.

E.7. Following transfection, incubate the cells at 37 °C, 120 rpm and in a humidified atmosphere with 8% CO_2_ in an orbital shaker incubator.

E.8. At 48 h post-transfection, add 5 mL of sodium heparin solution (Hospira Prod. Farm. y Hosp., S.L.) to the cells.

Note 5: We typically use a sterile sodium heparin solution with 5000 IU/mL (equivalent to 50 mg/mL heparin with a molecular weight of 12,000–15,000 Da), derived from porcine intestinal mucosa and suitable for cell culture.

E.9. Incubate the cells in an orbital shaker for additional 5–10 days at 37 °C, 120 rpm and in a humidified atmosphere with 8% CO_2._

Note 6: The optimal expression time to obtain maximum protein yield should be determined experimentally for each protein of interest.

E.10. Harvest the cells by centrifugation at 3000× *g* for 5 min. Use the supernatant immediately for the downstream protein purification or store it at −80 °C until further use.

### 2.3. Optimized Protein Purification Protocol

P.1. Defrost the medium (if necessary) and add a cocktail of EDTA-free protease inhibitors (highly recommendable, depending on the protein).

Note 7: The protocol of purification described here was optimized for the purification of CPZ, and, therefore, for other heparin affinity proteins, additional steps can be optimized to assure maximum protein recovery.

Note 8: It is advisable to run an SDS-PAGE of the conditioned medium prior to purification to confirm the expression of the target protein.

#### 2.3.1. Purification Step-1

Heparin affinity purification step using a Heparin HyperD^®^ resin (PALL Life Sciences, Port Washington, NY, USA). We recommend following the manufacturer’s protocol with minor modifications as follows.

P.2 Equilibrate 10.0 mL of heparin affinity resin per liter of culture medium using 3 column volumes of resin equilibration buffer (Buffer 1: 100 mM Tris-HCl, 100 mM NaCl, pH 7.4).

P.3 Flow the clarified conditioned medium containing the recombinant protein and discard the flow-through.

P.4 Wash the resin with 2 column volumes (20 mL) of equilibration Buffer 1.

P.5 Elute the recombinant protein by applying an increasing gradient of NaCl up to 1.5 M using the same equilibration buffer. Eluting buffer could be increased stepwise and applied to the column (e.g., 0.2, 0.3, 0.4, 0.5, 0.6, 0.7, 0.8, 0.9 and 1.0 M NaCl). Alternatively, a linear NaCl gradient could be applied using an automated FPLC system. Collect a 10 µL sample of each eluate and analyze them by SDS-PAGE.

#### 2.3.2. Purification Step-2

P.6 Equilibrate 5.0 mL of Strep-tag affinity resin (IBA-Lifesciences, Gottingen, Germany) per liter of culture medium by washing three times with resin equilibration buffer (Buffer 2: 100 mM Tris-HCl, 150 mM NaCl, pH 8.0).

P.7 Load the purest samples from step P.5 into the column and collect 10 μL of each eluted fraction. If small aggregates are present, the protein can be filtered through a 0.22 µm filter.

P.8 Wash the resin with 25 mL of equilibration Buffer 2.

P.9 Elute the recombinant protein with 12 column volumes of elution buffer (100 mM Tris-HCl, 150 mM NaCl, pH 8.0 and 2.5 mM d-desthiobiotin (IBA-Lifesciences Gottingen, Germany)). Collect a 10 µL sample of each eluate and analyze by SDS-PAGE.

#### 2.3.3. Purification Step-3

P.10 Equilibrate the size exclusion chromatography column with gel filtration buffer (25 mM Tris-HCl, 150 mM NaCl, pH 8.0).

P.11 Load the purest samples from step P.9 into the column and collect 10 μL of each eluted fraction. If small aggregates are present, the protein can be filtered through a 0.22 µm filter.

P.12 Run samples from this step on an SDS-PAGE and Coomassie stain for analysis. In this step, all the purest fractions can be pooled and stored at −80 °C.

Note 9: This purification step is useful to perform a desalting and buffer exchange of our sample. It is also useful to remove possible macromolecules and small contaminants present in the sample after the second purification step (e.g., d-desthiobiotin).

Note 10. The quality of our protein can also be tested by assessing its biological activity. It is also highly advisable to monitor the progress of the purification by measuring the activity (e.g., enzymatic activity) of our sample in the expression medium and after each purification step.

### 2.4. Cytotoxicity Experiments

For the cytotoxicity experiments, HEK 293F cells were seeded into 96-well plates using per well 3.0 × 10^3^ cells prepared in 200 µL of FreeStyle 293 expression medium. Three wells were filled only with 200 µL of FreeStyle 293 expression medium and were used as plate blank (see below). The 96-well plates were incubated for 24 h before sodium heparin (Hospira Prod. Farm. y Hosp, S.L.) was added at the indicated concentration (ranging from 0 to 2000 IU/mL); each condition was tested in triplicate. The growth inhibitory effect was measured after 24 and 72 h of treatment by the XTT assay [26,27]. The XTT assay is based on the ability of metabolically active cells (i.e., viable cells) to cleave the tetrazolium salt XTT into the orange formazan dye. The amount of formazan formed can be evaluated by reading the absorbance at a wavelength between 450 and 500 nm. Briefly, aliquots of 20 μL of XTT solution (2,3-bis-(2-methoxy-4-nitro-5-sulfophenyl)-2H-tetrazolium-5-carboxanilide) were added to each well. After 4 h, the color formed was quantified by a spectrophotometric plate reader (Perkin Elmer Victor3 V, Waltham, MA, USA) at 490 nm. Cell cytotoxicity was evaluated in terms of cell growth inhibition in treated cultures and expressed as % of the control condition (i.e., 0 IU/mL sodium heparin). For this calculation, the absorbance of wells containing only the XTT reagent and FreeStyle 293 expression medium (the plate blank) was subtracted from all wells. The results were expressed as the percentage of cell viability relative to control cells (which were considered 100%) and used to plot dose–response curves.

### 2.5. Enzyme Activity Assays

The carboxypeptidase activity of CPZ was assayed with the fluorescent substrate dansyl-Phe-Ala-Arg as previously described [23]. In brief, a 100 µL reaction mix, containing 0.2 mM of dansyl-Phe-Ala-Arg in 100 mM Tris-acetate, pH 7.5, 100 mM NaCl was incubated with a final concentration of 100 nM of CPZ for 60 min at 37 °C. After incubation, reactions were stopped by adding 50 µL of 0.5 M HCl. Then, 1 mL of chloroform was added to each reaction and the tubes were gently mixed and centrifuged for 2 min at 300× *g*. After centrifugation, 0.5 mL of the chloroform phase was transferred to new tubes and completely dried overnight at 25 °C. Finally, dried samples containing mainly the product generated in the enzymatic reaction were resuspended with 200 µL of PBS containing 0.1% of Triton X-100. The amount of product generated was determined by fluorescence at 395 nm upon excitation at 350 nm, using a 96-well plate spectrofluorometer.

### 2.6. Statistical Analyses

To identify changes in protein expression levels between heparin-supplemented and control cultures as a function of time, the data (expressed as mean ± SEM) was analyzed using a two-way analysis of variance (ANOVA), followed by Sidak’s multiple comparison tests. These multiple comparisons were limited to comparing the mean protein levels in the presence and absence of heparin for the different time points, as this is the main interest of our study. To evaluate the effect of heparin on cell viability, a one-way ANOVA followed by Sidak’s multiple comparisons test was performed. In all cases, an α level (level of significance) of 5% was used. All statistical analyses were performed using GraphPad Prism version 6.01 [28].

## 3. Results

### 3.1. Heparin as an Additive to Enhance Recombinant Protein Expression

The recombinant production of ECM-bound molecules can present challenges, particularly in the medium-to-large scale necessary for biopharmaceutical applications. ECM binding is typically mediated by their interactions with heparan sulfate proteoglycans (HSP), which are highly sulfated polysaccharides with structural similitude to the anticoagulant molecule heparin [15]. Thus, it is not surprising that many of the ECM-bound proteins are traditionally classified as heparin-binding proteins. Upon binding to heparin, these secreted molecules are protected from aggregation and proteolytic degradation, which often leads to increased yields of the full-active protein in the extracellular culture medium [13,29].

**Figure 1:**
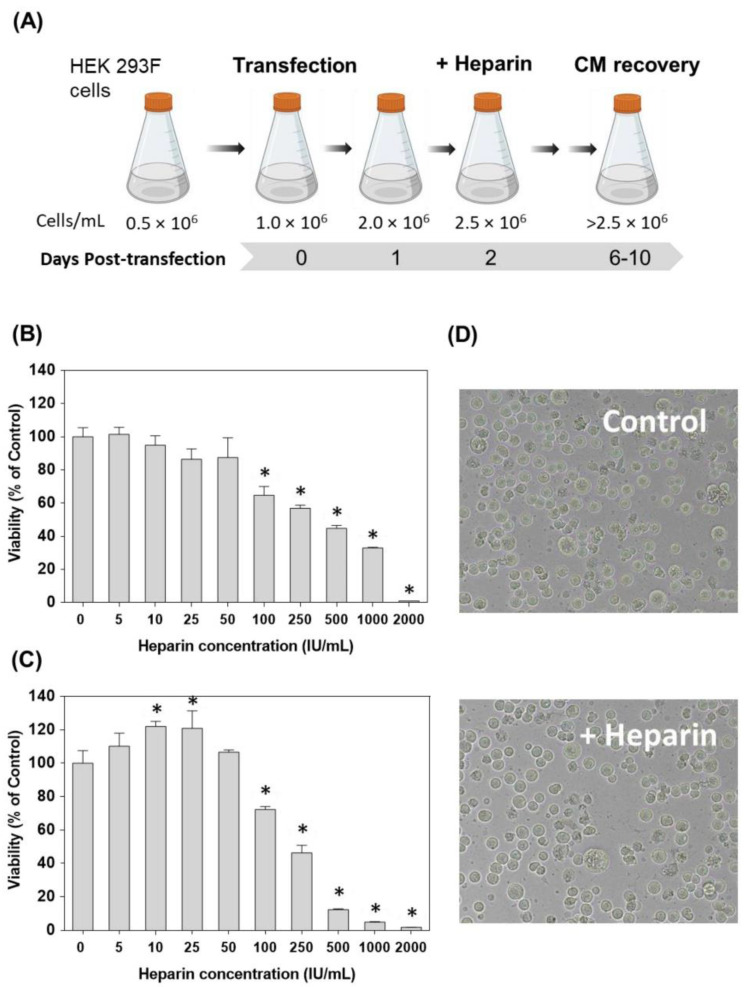
Schematic representation of the optimized pipeline for the production of ECM proteins with affinity by heparin. (**A**) HEK 293F cells were seeded at a cell density of 0.5 × 10^6^ cells/mL. After 24 h, cells were transfected with the DNA/PEI mix, and after 48 h, the cell culture was supplemented with 50 IU/mL of sodium heparin to enhance protein production. After the appropriate incubation time (typically between 7 and 10 days), the cell culture can be recovered, centrifuged and the conditioned medium (CM) used for protein purification. (**B**,**C**) The cytotoxicity of sodium heparin against HEK 293F cells was evaluated after (**B**) 24 and (**C**) 72 h of treatment with heparin at a concentration up to 2000 IU/mL. Fifty IU/mL was the highest concentration of sodium heparin that did not cause notable cytotoxicity against HEK 293F cells. (**D**) Representative images of HEK 293F cells after 72 h incubation, with or without 50 IU/mL of sodium heparin. In (**B**) and (**C**), data are shown as mean ± SD of three independent experiments. One-way ANOVA followed by Sidak’s multiple comparisons test to evaluate the effect of heparin on cell viability. The asterisk represents a significant difference in means between heparin treatment and control cells when an α of 0.05 is considered.

This approach outlines a detailed strategy for the transient expression of ECM-binding proteins with affinity for heparin in HEK 293F cells (Figure 1A and Materials and Methods section). In our experience, enhanced amounts of recombinant protein expression were typically obtained after supplementation with 50 IU/mL of sodium heparin two days after transfection. At this concentration, no toxic effects of heparin on HEK 293F cells were observed up to 72 h (Figure 1B–D).

### 3.2. Enhanced Expression of Biomedically Relevant ECM/Heparin-Binding Proteins by Heparin Supplementation

**Figure 2:**
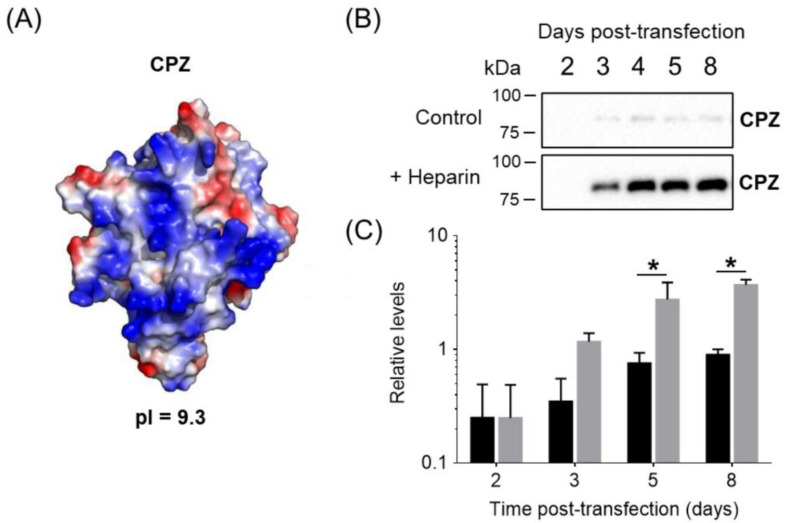
Enhanced expression of human CPZ in HEK 293F cells after heparin supplementation. (**A**) Electrostatic surface potential distribution of the catalytic domain of human CPZ (AlphaFold model AF-Q66K79-F1). Blue and red indicate positive and negative charge potential, respectively. The isoelectric point (pI) of the full-length CPZ is indicated. (**B**) Representative immunoblots of the human CPZ expression over time in HEK 293F cells in the absence (black bars) and presence (grey bars) of heparin as additive (50 IU/mL). CPZ levels were determined using an anti-Strep-tag antibody. (**C**) Representative relative expression levels of human CPZ over time in HEK 293F cells. Values were normalized to the maximum CPZ signal detected in the conditioned medium in absence of heparin. Values are mean ± SEM of 3 independent experiments. Two-way ANOVA was performed, followed by Sidak’s multiple comparison test, which was limited to comparing the mean protein levels in the presence and absence of heparin for the different time points. The two-way ANOVA suggested that CPZ expression levels depended both on the presence/absence of heparin and the days of expression. The asterisk represents a significant difference in means between absence and presence of heparin when an α of 0.05 is considered.

Using this expression pipeline, we successfully expressed the human carboxypeptidase Z (CPZ), which is a human ECM-bound metalloenzyme involved in neuropeptide processing. This protein is extremely difficult to express in the absence of heparin supplementation [23]. In the absence of heparin, most of the produced CPZ in adherent cell cultures is bound to the ECM due to its heparin-binding properties (Figure 2A) [23,29]. As shown in Figure 2B, heparin addition after two days post-transfection resulted in a notable accumulation of the enzyme in the extracellular medium that was up to five-fold higher compared to the unsupplemented expression.

**Figure 3:**
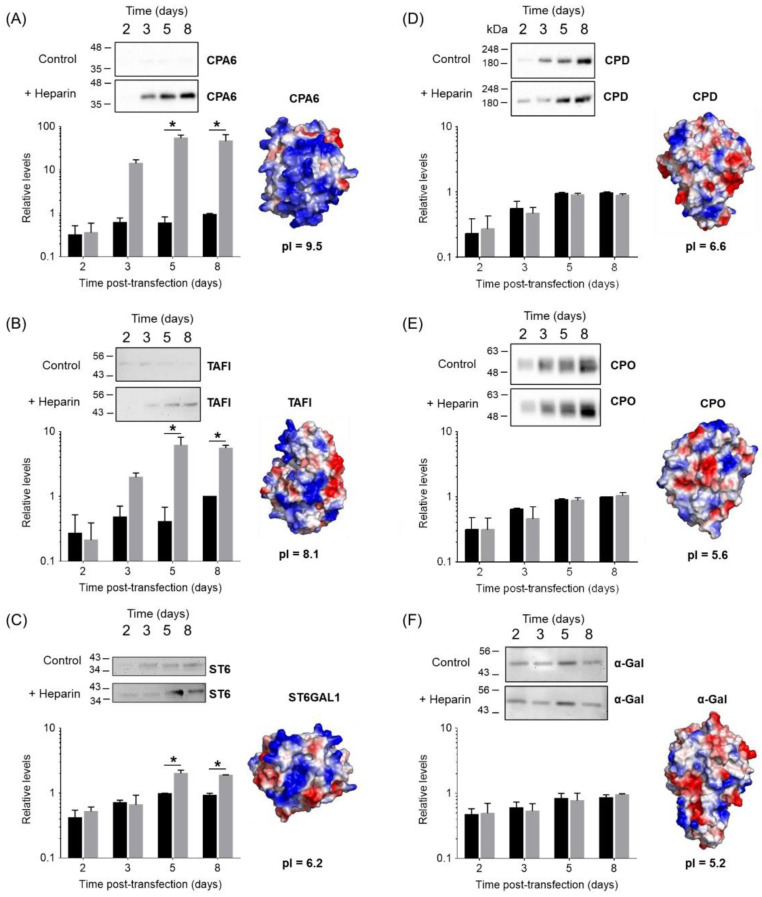
Comparative expression profiles of a set of biomedically relevant enzymes upon heparin supplementation. Immunoblots and relative expression levels were determined for three different heparin-binding proteins: (**A**) CPA6, (**B**) TAFI and (**C**) ST6GAL1. The expression was evaluated in the absence (black bars) or in the presence (grey bars) of 50 IU/mL of sodium heparin added 72 h after transfection, as described in the experimental section. The expression profiles of (**D**) CPD, (**E**) CPO and (**F**) α-galactosidase (α-gal) were analyzed as control proteins. In all cases, the values were normalized to the maximum protein signal detected in the conditioned medium in absence of heparin. Values are mean ± SEM of 3 independent experiments. In all cases, structural models showing the electrostatic surface potential distribution are presented. The coordinates used are: CPA6 (AlphaFold model AF-Q8N4T0-F1), TAFI (PDB code 3LMS), ST6GAL1 (PDB code 4JS1), CPD domain II (AlphaFold model AF-O75976-F1), CPO (PDB code 5MRV) and α-gal (6IBR). All the structural representations were generated with Pymol [36]. Two-way ANOVA was performed, followed by Sidak’s multiple comparison test, which was limited to comparing the mean protein levels in the presence and absence of heparin for the different time points. The two-way ANOVA suggested that CPA6, TAFI and ST6GAL1 expression levels depended both on the presence/absence of heparin and the days of expression. For CPD and CPO, the two-way ANOVA indicates that only the time of expression significantly influences the levels of expression of these proteins. Meanwhile, α-gal expression levels are independent of the presence/absence of heparin and the expression time. The asterisk represents a significant difference in means between absence and presence of heparin when an α of 0.05 is considered.

To demonstrate the robustness of our approach on different ECM-binding proteins, we further tested the expression of six additional biomedically relevant enzymes (Figure 3). Three of such proteins (TAFI, CPA6 and ST6GAL1) were expected to have heparin/ECM-binding properties based on previous findings (Figure 3A–C) [30,31,32,33,34,35]. After heparin supplementation, CPA6 protein levels in the conditioned medium were increased about 55-fold after 5 days post-transfection. Similarly, in the case of TAFI, the amount of secreted protein raised to 6-fold after 5 days post-transfection as a result of heparin addition. The smallest improvement was observed for ST6GAL1, for which only a 2-fold enhancement of protein levels was achieved. Interestingly, ST6GAL1 displays a very low pI in comparison to the other four heparin-binding proteins tested. As expected, our approach did not increase the extracellular levels of other proteins without heparin affinity properties, such as human CPD [22], carboxypeptidase O [24] or human α-galactosidase (α-gal) [25] (Figure 3E,F).

### 3.3. Our Approach Is Compatible with a Straightforward Purification of ECM/Heparin-Binding Proteins

**Figure 4:**
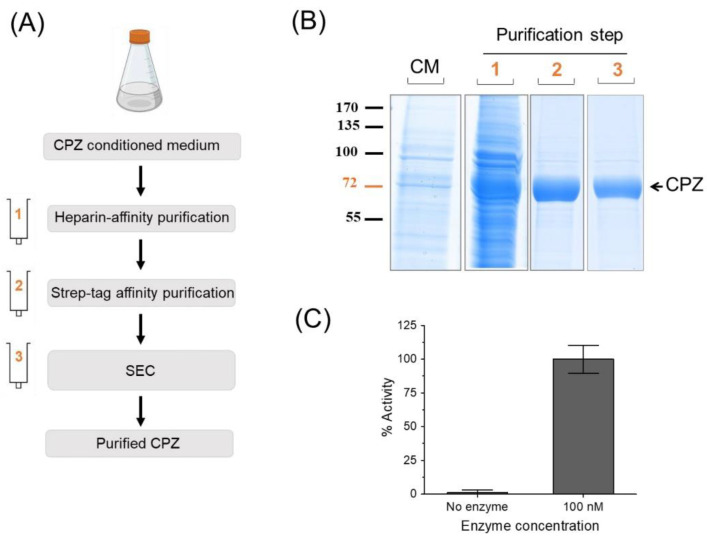
Purification of human CPZ. (**A**) Scheme showing the protocol followed for the purification of CPZ. For protein purification, the extracellular medium was collected after 8 days post-transfection and the recombinant protein was purified in three purification steps: (I) heparin affinity chromatography, (II) affinity chromatography using Strep-tag purification resin and (III) size exclusion chromatography (SEC), a protocol optimized previously by our group [23]. (**B**) An aliquot of the initial conditioned medium, as well as from the elution of each purification step, were visualized on SDS-PAGE by Coomassie staining. (**C**) The purified protein shows the carboxypeptidase activity against dansyl-Phe-Ala-Arg, a fluorescent CPB-like peptide substrate [23,39]. The activity values were normalized to the maximum activity detected in the presence of the enzyme. Values are mean ± SEM of 3 independent reactions.

As described above, a typical procedure for the expression of CPZ was applied. After optimizing the expression conditions, we produced large amounts (2 L) of conditioned medium to test whether the expressed protein is of high quality and suitable for protein purification. After 8 days post-transfection, the conditioned medium from the culture was recovered and CPZ purified through an optimized three-step purification protocol, involving two affinity and one size exclusion chromatographies (Figure 4A). Figure 4B displays an SDS-PAGE showing the presence of CPZ in the conditioned medium and the purity of CPZ after each purification step. Heparin has high negative charge [37], which can affect the electrostatic binding of proteins to ionic exchangers during their purification. In order to determine the effect of this additive on protein purification, we performed a first affinity purification of CPZ using heparin as a ligand. Interestingly, even though sodium heparin was added during protein expression to solubilize the secreted protein, this additive did not interfere with a typical heparin affinity purification. After the first step, eluted fractions containing CPZ protein were pooled and loaded into a Strep-tag affinity resin and further purified using size exclusion chromatography (SEC) (Figure 4B). The final purified protein showed a high purity (Figure 4B). Approximately, a yield of 2–3 mg of active protein per liter of cell culture was obtained (Figure 4C). In the absence of heparin, such challenges can result in low yields of protein production or even in the absence of detectable levels of protein secretion [23,38].

### 3.4. Considerations for Selecting ECM/Heparin-Binding Proteins

**Figure 5:**
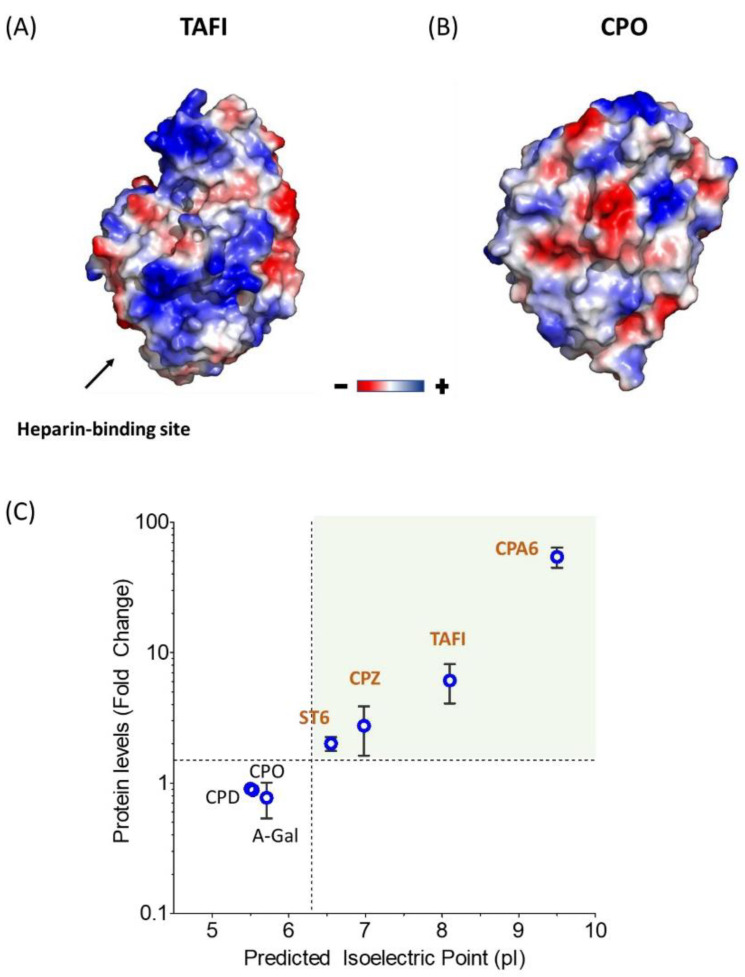
Correlation between protein pI and the heparin-driven enhancement of protein expression. (**A**,**B**) Comparative electrostatic surface potential distribution between the catalytic domain of (**A**) TAFI and (**B**) CPO, as representative examples of heparin-binding and non-heparin-binding proteins. The putative heparin-binding region of TAFI is indicated as “heparin-binding site” [30]. By contrast, CPO lacks the typical surface clustering of basic residues necessary for heparin binding. (**C**) Correlation plot showing the expression fold change upon heparin supplementation and the predicted protein pI for all the expression constructs evaluated in this report. Note that higher pIs are associated with an increased enhancement in protein production by the cells upon heparin addition. The upper right quadrant (highlighted in green) encircles the ECM/heparin-binding proteins that were produced at levels ≥ 1.5-fold, all of them with a pI higher than 6.3.

Surface electrostatics clearly plays a major role in heparin/ECM–protein interactions [40]. Typically, these proteins are enriched in basic amino acids such as arginine and lysine. As shown in Figure 2A, the modeled structure of the catalytic domain of human CPZ serves as a prototypical example of the characteristic electrostatic surface potential distribution found in heparin-binding proteins. This model clearly displays a large number of basic residues (i.e., Arg and Lys) clustered at a highly polarized protein surface, which mediate ECM binding [29]. Next, we performed an accurate analysis of all members of the M14 family of proteases. Similar electrostatic surface potential distribution patterns were observed for other enzymes from this protein family such as carboxypeptidase A3 (CPA3), CPA6 or TAFI, all of them with proven ECM/heparin-binding properties. Overall, these proteins have a marked bias towards a basic amino acid content and thus display the most basic isoelectric points (pIs) among all members of the M14 family of proteases (Table S1) [41]. It is also common in these proteins for the local arrangement at the surface of basic amino acids as heparin-binding sites and a clear polarization of basic residues at the protein surface (Figure 5A) [30,41,42]. Such clustering of basic amino acids rarely occurs in other extracellular MCPs without heparin-binding properties (Figure 5B).

In an effort to identify common physicochemical features that affect protein expression, we analyzed the relationship between the enhancement in protein expression observed in Figure 3 and the pI of these proteins. When plotting the expression level fold change upon heparin supplementation versus protein pI, a clear correlation between these two variables is observed (Figure 5C). This finding suggests that the overall pI of a protein is one of the key parameters that can be used to predict the particular response of a protein to heparin supplementation. More interestingly, this correlation also provides a reference for selecting novel protein targets that would benefit from the present protein expression approach.

## 4. Discussion

The initial and often most used and challenging step necessary for the bioproduction of pharmaceutically relevant proteins is their recombinant protein expression. Here, we described a straightforward and efficient protein expression pipeline that was able to provide a robust enhancement of the recombinant expression levels for proteins with proven ECM/heparin-binding properties. The proof of concept experiments on CPZ, CPA6, TAFI and ST6GAL1 show the advantages of heparin supplementation, which further demonstrates the robustness and suitability of this approach for the production of novel pharmaceutical protein targets. These selected proteins are novel enzymes with relevant physiological functions. For instance, CPZ is a secreted zinc-containing exopeptidase that functions in the extracellular processing of neuropeptides and growth factors [23]. CPA6 is also an ECM-bound enzyme with carboxypeptidase activity. Deletion of part of the CPA6 gene has been associated with the development of the Duane syndrome, which suggests that this enzyme plays a pivotal role in the migration and axonal guidance during embryonic development [13]. TAFI is an important metalloenzyme involved in coagulation regulation and forms a molecular link between coagulation and fibrinolysis [42]. ST6GAL1 is a key player in cancer development since it catalyzes the addition of α2,6-linked sialic acids to terminal N-glycans [43]. ST6GAL1 upregulation has been associated with numerous types of cancer including pancreatic, prostate, breast and ovarian tumors [43].

Notably, heparin supplementation led to an increase in the extracellular protein levels between 2- and 55-fold when compared to the expression in the absence of heparin (see Figure 2 and Figure 3). It has been shown that the formation of heparin-bound protein complexes strongly correlates with protein stabilization and solubilization [15]. This might explain the observed increase in the recombinant protein yields during expression. Furthermore, we showed that the improvement in protein yield is particularly good for proteins with basic pIs, observing a strong correlation between pI and the fold-change improvement in expression levels induced by heparin (Figure 5C). We evaluated our approach using suspension HEK 293F cells, one of the most utilized expression hosts for recombinant protein expression. However, in addition to HEK 293-derived cell lines, our approach can be applied to other common mammalian protein expression systems, such as CHO cells [1]. In addition, this approach could be extended to other common expression systems that are able to express recombinant proteins in the extracellular media. The latter might include insect cell expression host systems (e.g., Sf9 cells, Sf21, Tn-368 and High-Five™) or yeast expression systems (e.g., *Saccharomyces cerevisiae* or PichiaPink) [1]. In summary, it is possible that our heparin supplementation approach and the findings presented here can be extended to multiple expression platforms with similar results.

Previous studies have demonstrated that heparin strongly binds to a wide range of pharmaceutically relevant proteins [15,42,44]. Thus, it is not surprising that several ECM-bound proteins can be released from the matrix to the culture medium upon heparin binding [13,23,29]. Moreover, heparin and heparin derivatives are commonly used in heparin affinity chromatography as a very effective and simple method to purify a wide range of proteins [45,46]. We have also demonstrated that heparin supplementation is compatible with a purification workflow that includes an affinity purification step that uses heparin as a ligand. Using CPZ, a proven case of a difficult-to-produce protein, we demonstrated that this workflow is able to purify proteins with a high degree of purity and in the active form (Figure 4B), obtaining the highest protein yield reached for CPZ reported so far (about 2–3 mg/L of cell culture). The presence of heparin in the cell culture media does not interfere with the binding of proteins to a heparin affinity resin. Furthermore, such a purification step is a good tool to remove the additive from the recombinant protein solution.

Previous attempts to express CPZ using either insect cells or mammalian cells resulted in extremely low levels of CPZ expression in the cell culture media and, as a result, failed protein purification attempts [23,38,47]. Similarly, the expression levels of several pharmaceutically relevant proteins are still too low or extremely difficult to express. Thus, our approach can be very useful to produce ECM proteins with affinity by heparin as well as other secreted basic proteins for which their expression remained elusive. To our knowledge, this is the first comprehensive study of the use of heparin supplementation as an enhancer of the protein expression of ECM/heparin-binding proteins.

## Data Availability

The authors declare that all the data presented in this study are available in the paper or in the supplementary information. Additional raw data supporting the findings of this study are available from the corresponding author upon reasonable request.

## Supplementary Materials

The following supporting information can be downloaded at: https://www.mdpi.com/article/10.3390/pharmaceutics14102138/s1, Table S1: ECM/heparin-binding properties of human metallocarboxypeptidases from the M14 family of proteases.

## References

[1] Tripathi N.K., Shrivastava A. (2019). Recent Developments in Bioprocessing of Recombinant Proteins: Expression Hosts and Process Development. Front. Bioeng. Biotechnol..

[2] Khan K.H. (2013). Gene Expression in Mammalian Cells and its Applications. Adv. Pharm. Bull..

[3] Dumont J.A., Euwart D., Mei B., Estes S., Kshirsagar R.R. (2015). Human cell lines for biopharmaceutical manufacturing: History, status, and future perspectives. Crit. Rev. Biotechnol..

[4] Schmidt F.R. (2004). Recombinant expression systems in the pharmaceutical industry. Appl. Microbiol. Biotechnol..

[5] Li Z.-M., Fan Z.-L., Wang X.-Y., Wang T.-Y. (2022). Factors Affecting the Expression of Recombinant Protein and Improvement Strategies in Chinese Hamster Ovary Cells. Front. Bioeng. Biotechnol..

[6] Portolano N., Watson P.J., Fairall L., Millard C., Milano C.P., Song Y., Cowley S., Schwabe J.W.R. (2014). Recombinant Protein Expression for Structural Biology in HEK 293F Suspension Cells: A Novel and Accessible Approach. J. Vis. Exp..

[7] Walsh G. (2018). Biopharmaceutical benchmarks 2018. Nat. Biotechnol..

[8] Thoring L., Dondapati S.K., Stech M., Wüstenhagen D.A., Kubick S. (2017). High-yield production of “difficult-to-express” proteins in a continuous exchange cell-free system based on CHO cell lysates. Sci. Rep..

[9] Malm M., Kuo C.-C., Barzadd M.M., Mebrahtu A., Wistbacka N., Razavi R., Volk A.-L., Lundqvist M., Kotol D., Tegel H. (2022). Harnessing secretory pathway differences between HEK293 and CHO to rescue production of difficult to express proteins. Metab. Eng..

[10] Sohail A.A., Gaikwad M., Khadka P., Saaranen M.J., Ruddock L.W. (2020). Production of Extracellular Matrix Proteins in the Cytoplasm of E. coli: Making Giants in Tiny Factories. Int. J. Mol. Sci..

[11] Frantz C., Stewart K.M., Weaver V.M. (2010). The extracellular matrix at a glance. J. Cell Sci..

[12] Yue B. (2014). Biology of the Extracellular Matrix. J. Glaucoma.

[13] Lyons P., Callaway M.B., Fricker L.D. (2008). Characterization of Carboxypeptidase A6, an Extracellular Matrix Peptidase. J. Biol. Chem..

[14] Karamanos N.K., Theocharis A.D., Piperigkou Z., Manou D., Passi A., Skandalis S.S., Vynios D.H., Orian-Rousseau V., Ricard-Blum S., Schmelzer C.E. (2021). A guide to the composition and functions of the extracellular matrix. FEBS J..

[15] Capila I., Linhardt R.J. (2002). Heparin-protein interactions. Angew. Chem..

[16] Meneghetti M.C.Z., Hughes A., Rudd T., Nader H.B., Powell A.K., Yates E.A., Lima M.A. (2015). Heparan sulfate and heparin interactions with proteins. J. R. Soc. Interface.

[17] Wilgus T.A. (2012). Growth Factor–Extracellular Matrix Interactions Regulate Wound Repair. Adv. Wound Care.

[18] Zhu J., Clark R.A. (2014). Fibronectin at Select Sites Binds Multiple Growth Factors and Enhances their Activity: Expansion of the Collaborative ECM-GF Paradigm. J. Investig. Dermatol..

[19] Ling L., Camilleri E.T., Helledie T., Samsonraj R.M., Titmarsh D.M., Chua R.J., Dreesen O., Dombrowski C., Rider D.A., Galindo M. (2015). Effect of heparin on the biological properties and molecular signature of human mesenchymal stem cells. Gene.

[20] Xu X., Dai Y. (2009). Heparin: An intervenor in cell communication. J. Cell. Mol. Med..

[21] Huang J., Zhang L., Wan D., Zhou L., Zheng S., Lin S., Qiao Y. (2021). Extracellular matrix and its therapeutic potential for cancer treatment. Signal Transduct. Target. Ther..

[22] Garcia-Pardo J., Tanco S., Díaz L., Dasgupta S., Fernandez-Recio J., Lorenzo J., Aviles F.X., Fricker L.D. (2017). Substrate specificity of human metallocarboxypeptidase D: Comparison of the two active carboxypeptidase domains. PLoS ONE.

[23] Garcia-Pardo J., Tanco S., Garcia-Guerrero M.C., Dasgupta S., Avilés F.X., Lorenzo J., Fricker L.D. (2020). Substrate Specificity and Structural Modeling of Human Carboxypeptidase Z: A Unique Protease with a Frizzled-Like Domain. Int. J. Mol. Sci..

[24] Garcia-Guerrero M.C., Garcia-Pardo J., Berenguer E., Fernandez-Alvarez R., Barfi G.B., Lyons P.J., Aviles F.X., Huber R., Lorenzo J., Reverter D. (2018). Crystal structure and mechanism of human carboxypeptidase O: Insights into its specific activity for acidic residues. Proc. Natl. Acad. Sci. USA.

[25] Corchero J.L., Mendoza R., Lorenzo J., Rodríguez-Sureda V., Domínguez C., Vázquez E., Ferrer-Miralles N., Villaverde A. (2011). Integrated approach to produce a recombinant, his-tagged human α-galactosidase a in mammalian cells. Biotechnol. Prog..

[26] Núñez C., Oliveira E., García-Pardo J., Diniz M., Lorenzo J., Capelo J.L., Lodeiro C. (2014). A novel quinoline molecular probe and the derived functionalized gold nanoparticles: Sensing properties and cytotoxicity studies in MCF-7 human breast cancer cells. J. Inorg. Biochem..

[27] Roehm N.W., Rodgers G.H., Hatfield S.M., Glasebrook A.L. (1991). An improved colorimetric assay for cell proliferation and viability utilizing the tetrazolium salt XTT. J. Immunol. Methods.

[28] (2012).

[29] Novikova E.G., Reznik S.E., Varlamov O., Fricker L.D. (2000). Carboxypeptidase Z Is Present in the Regulated Secretory Pathway and Extracellular Matrix in Cultured Cells and in Human Tissues. J. Biol. Chem..

[30] Sanglas L., Valnickova Z., Arolas J.L., Pallares I., Guevara T., Solà M., Kristensen T., Enghild J.J., Aviles F.X., Gomis-Ruth F.X. (2008). Structure of Activated Thrombin-Activatable Fibrinolysis Inhibitor, a Molecular Link between Coagulation and Fibrinolysis. Mol. Cell.

[31] Lyons P.J., Fricker L.D. (2010). Substrate Specificity of Human Carboxypeptidase A6. J. Biol. Chem..

[32] Lyons P.J., Ma L.-H., Baker R., Fricker L.D. (2010). Carboxypeptidase A6 in Zebrafish Development and Implications for VIth Cranial Nerve Pathfinding. PLoS ONE.

[33] Salzmann A., Guipponi M., Lyons P.J., Fricker L.D., Sapio M., Lambercy C., Buresi C., Bencheikh B.O.A., Lahjouji F., Ouazzani R. (2011). Carboxypeptidase A6 gene (CPA6) mutations in a recessive familial form of febrile seizures and temporal lobe epilepsy and in sporadic temporal lobe epilepsy. Hum. Mutat..

[34] Sanglas L., Arolas J.L., Valnickova Z., Aviles F.X., Enghild J.J., Gomis-Rüth F.X. (2010). Insights into the molecular inactivation mechanism of human activated thrombin-activatable fibrinolysis inhibitor, TAFIa. J. Thromb. Haemost..

[35] Schwarting G.A., Gajewski A., Carroll P., Dewolf W.C. (1987). Inhibition of ganglioside sialyltransferase activity and stimulation of neutral glycolipid exocytosis by heparin. Arch. Biochem. Biophys..

[36] Schrödinger L. The PyMOL Molecular Graphics System, 2.0. http://www.pymol.org/pymol.

[37] Weiss R.J., Esko J.D., Tor Y. (2017). Targeting heparin and heparan sulfate protein interactions. Org. Biomol. Chem..

[38] Novikova E.G., Fricker L.D. (1999). Purification and Characterization of Human Metallocarboxypeptidase Z. Biochem. Biophys. Res. Commun..

[39] Kapiloff M., Strittmatter S.M., Fricker L.D., Snyder S.H. (1984). A fluorometric assay for angiotensin-converting enzyme activity. Anal. Biochem..

[40] Xu D., Esko J.D. (2014). Demystifying Heparan Sulfate–Protein Interactions. Annu. Rev. Biochem..

[41] Arolas J., Vendrell J., Aviles F., Fricker L. (2007). Metallocarboxypeptidases: Emerging Drug Targets in Biomedicine. Curr. Pharm. Des..

[42] Anand K., Pallares I., Valnickova Z., Christensen T., Vendrell J., Wendt K.U., Schreuder H.A., Enghild J.J., Aviles F.X. (2008). The Crystal Structure of Thrombin-activable Fibrinolysis Inhibitor (TAFI) Provides the Structural Basis for Its Intrinsic Activity and the Short Half-life of TAFIa. J. Biol. Chem..

[43] Garnham R., Scott E., Livermore K.E., Munkley J. (2019). ST6GAL1: A key player in cancer (Review). Oncol. Lett..

[44] Ghiselli G. (2019). Heparin Binding Proteins as Therapeutic Target: An Historical Account and Current Trends. Medicines.

[45] Bolten S.N., Rinas U., Scheper T. (2018). Heparin: Role in protein purification and substitution with animal-component free material. Appl. Microbiol. Biotechnol..

[46] Xiong S., Zhang L., He Q.-Y. (2008). Fractionation of Proteins by Heparin Chromatography. 2D PAGE Sample Prep. Fractionation.

[47] Song L., Fricker L.D. (1997). Cloning and Expression of Human Carboxypeptidase Z, a Novel Metallocarboxypeptidase. J. Biol. Chem..

