# Supplementary material for: Enhanced Production of ECM Proteins for Pharmaceutical Applications Using Mammalian Cells and Sodium Heparin Supplementation
Source: Pharmaceutics. 2022 Oct 8;14(10):2138. doi: 10.3390/pharmaceutics14102138 (PMC9609459; doi:10.3390/pharmaceutics14102138)
Supplement: Supplementary file 1 [file pharmaceutics-14-02138-s001.zip › pharmaceutics-1869648-supplementary.pdf]

pharmaceutics

MDPI

SUPPLEMENTARY INFORMATION

Enhanced production of ECM proteins for pharmaceutical applications using mammalian cells and sodium heparin supplementation

Javier Garcia-Pardo *, Sergi Montané, Francesc Xavier Avilés, Sebastian Tanco and Julia Lorenzo *

Institut de Biotecnologia i Biomedicina and Departament de Bioquímica i Biologia Molecular, Universitat Autònoma de Barcelona, 08193 Bellaterra, Barcelona, Spain

*Correspondence: javiergarciapardo@msn.com (J.G-P.); julia.lorenzo@uab.cat (J.L.); Tel.: +34-93-586-8936 (J.G-P); +34-93-586-8957 (J.L)

SUPPLEMENTARY TABLE

Table S1. ECM/heparin-binding properties of human metallocarboxypeptidases from the M14 family of proteases.

	Uniprot Code	Protein Name	Localization	pI*	ECM/Heparin-binding	Ref.
M14A Subfamily	P15085	Carboxypeptidase A1 (CPA1)	Extracellular	5.5 / 5.9	Unknown	[1]
	P48052	Carboxypeptidase A2 (CPA2)	Extracellular	5.5 / 6.3	Unknown	[1]
	P15086	Carboxypeptidase B (CPB)	Extracellular	6.2 / 6.7	Unknown	[1]
	P15088	Mast cell carboxypeptidase A (CPA3)	Extracellular	**9.2 / 9.5**	Unknown	[1]
	Q9UI42	Carboxypeptidase A4 (CPA4)	Extracellular	6.1 / 7.1	Unknown	[1,2]
	Q8WXQ2	Carboxypeptidase A5 (CPA5)	Unknown	6.0 / 5.8	Unknown	[1]
	Q8N4T0	Carboxypeptidase A6 (CPA6)	Extracellular	9.5 / 9.5	Yes	[3]
	Q96IY4	Carboxypeptidase B2 (TAFI)	Extracellular	7.7 / 8.1	Yes	[4]
	Q8IVL8	Carboxypeptidase O (CPO)	Extracellular	6.6	Unknown	[5]
M14B Subfamily	O75976	Carboxypeptidase D (CPD)	TGN / Extracellular	5.6	Unknown	[1,6]
	P14384	Carboxypeptidase M (CPM)	Cell membrane / Extracellular	6.7	Unknown	[1,7]
	P15169	Carboxypeptidase N catalytic chain (CPN)	Extracellular	6.9	Unknown	[1]
	P16870	Carboxypeptidase E (CPE)	Secretory vesicles	4.9	Unknown	[1,8-10]
	Q66K79	Carboxypeptidase Z (CPZ)	Extracellular	8.3 / 9.3	Yes	[11-17]
	Q8IUX7	Adipocyte enhancer-binding protein 1 (AEBP1)	Cytosolic	5.0	Unknown	[1]
	Q96SM3	Carboxypeptidase X1 (CPX1)	Cytosolic	6.2	Unknown	[1]

	Q8N436	Carboxypeptidase-like protein X2 (CPX2)	Cytosolic	6.4	Unknown	[1]
**M14D Subfamily members**	Q9UPW5	Cytosolic carboxypeptidase 1 (CCP1)	Cytosolic / Nuclear	5.8	Unknown	[18-20]
	Q5U5Z8	Cytosolic carboxypeptidase 2 (CCP2)	Cytosolic	9.1	Unknown	[18-20]
	Q8NEM8	Cytosolic carboxypeptidase 3 (CCP3)	Cytosolic	9.0	Unknown	[18-20]
	Q96MI9	Cytosolic carboxypeptidase 4 (CCP4)	Cytosolic / Nuclear	6.9	Unknown	[18-20]
	Q8NDL9	Cytosolic carboxypeptidase 5 (CCP5)	Cytosolic	9.3	Unknown	[18-20]
	Q5VU57	Cytosolic carboxypeptidase 6 (CCP6)	Cytosolic	5.8	Unknown	[18-20]

pI values were calculated with the ProtParam tool from Expasy (http://web.expasy.org/protparam/). When multiple values are presented, the first value indicates the pI of the proform and the second value indicates the pI of the active form. In the case of CPZ, the second pI value corresponds to the enzyme without the Frizzled-like domain. The proteins evaluated in the present study are highlighted in green. TGN, Trans-Golgi network.

SUPPLEMENTARY REFERENCES

1. Arolas, J.L.; Vendrell, J.; Aviles, F.X.; Fricker, L.D. Metallocarboxypeptidases: emerging drug targets in biomedicine. *Current pharmaceutical design* **2007**, *13*, 349-366, doi:10.2174/138161207780162980.
2. Tanco, S.; Zhang, X.; Morano, C.; Aviles, F.X.; Lorenzo, J.; Fricker, L.D. Characterization of the substrate specificity of human carboxypeptidase A4 and implications for a role in extracellular peptide processing. *The Journal of biological chemistry* **2010**, *285*, 18385-18396, doi:10.1074/jbc.M109.060350.
3. Lyons, P.J.; Callaway, M.B.; Fricker, L.D. Characterization of carboxypeptidase A6, an extracellular matrix peptidase. *The Journal of biological chemistry* **2008**, *283*, 7054-7063, doi:10.1074/jbc.M707680200.
4. Sanglas, L.; Valnickova, Z.; Arolas, J.L.; Pallares, I.; Guevara, T.; Sola, M.; Kristensen, T.; Enghild, J.J.; Aviles, F.X.; Gomis-Ruth, F.X. Structure of activated thrombin-activatable fibrinolysis inhibitor, a molecular link between coagulation and fibrinolysis. *Molecular cell* **2008**, *31*, 598-606, doi:10.1016/j.molcel.2008.05.031.
5. Garcia-Guerrero, M.C.; Garcia-Pardo, J.; Berenguer, E.; Fernandez-Alvarez, R.; Barfi, G.B.; Lyons, P.J.; Aviles, F.X.; Huber, R.; Lorenzo, J.; Reverter, D. Crystal structure and mechanism of human carboxypeptidase O: Insights into its specific activity for acidic residues. *Proceedings of the National Academy of Sciences of the United States of America* **2018**, *115*, E3932-E3939, doi:10.1073/pnas.1803685115.
6. Garcia-Pardo, J.; Tanco, S.; Diaz, L.; Dasgupta, S.; Fernandez-Recio, J.; Lorenzo, J.; Aviles, F.X.; Fricker, L.D. Substrate specificity of human metallocarboxypeptidase D: Comparison of the two active carboxypeptidase domains. *PloS one* **2017**, *12*, e0187778, doi:10.1371/journal.pone.0187778.
7. Reverter, D.; Maskos, K.; Tan, F.; Skidgel, R.A.; Bode, W. Crystal structure of human carboxypeptidase M, a membrane-bound enzyme that regulates peptide hormone activity. *Journal of molecular biology* **2004**, *338*, 257-269, doi:10.1016/j.jmb.2004.02.058.
8. Fricker, L.D. Carboxypeptidase E and the Identification of Novel Neuropeptides as Potential Therapeutic Targets. *Advances in pharmacology* **2018**, *82*, 85-102, doi:10.1016/bs.apha.2017.09.001.

9. Fricker, L.D. Activation and membrane binding of carboxypeptidase E. *Journal of cellular biochemistry* **1988**, *38*, 279-289, doi:10.1002/jcb.240380407.
10. Fricker, L.D. Carboxypeptidase E. *Annual review of physiology* **1988**, *50*, 309-321, doi:10.1146/annurev.ph.50.030188.001521.
11. Garcia-Pardo, J.; Tanco, S.; Garcia-Guerrero, M.C.; Dasgupta, S.; Aviles, F.X.; Lorenzo, J.; Fricker, L.D. Substrate Specificity and Structural Modeling of Human Carboxypeptidase Z: A Unique Protease with a Frizzled-Like Domain. *International journal of molecular sciences* **2020**, *21*, doi:10.3390/ijms21228687.
12. Novikova, E.; Fricker, L.D.; Reznik, S.E. Metallocarboxypeptidase Z is dynamically expressed in mouse development. *Mechanisms of development* **2001**, *102*, 259-262, doi:10.1016/s0925-4773(01)00306-9.
13. Novikova, E.G.; Fricker, L.D. Purification and characterization of human metallocarboxypeptidase Z. *Biochemical and biophysical research communications* **1999**, *256*, 564-568, doi:10.1006/bbrc.1999.0378.
14. Novikova, E.G.; Reznik, S.E.; Varlamov, O.; Fricker, L.D. Carboxypeptidase Z is present in the regulated secretory pathway and extracellular matrix in cultured cells and in human tissues. *The Journal of biological chemistry* **2000**, *275*, 4865-4870, doi:10.1074/jbc.275.7.4865.
15. Reznik, S.E.; Fricker, L.D. Carboxypeptidases from A to z: implications in embryonic development and Wnt binding. *Cellular and molecular life sciences : CMLS* **2001**, *58*, 1790-1804, doi:10.1007/PL00000819.
16. Song, L.; Fricker, L.D. Cloning and expression of human carboxypeptidase Z, a novel metallocarboxypeptidase. *The Journal of biological chemistry* **1997**, *272*, 10543-10550, doi:10.1074/jbc.272.16.10543.
17. Xin, X.; Day, R.; Dong, W.; Lei, Y.; Fricker, L.D. Cloning, sequence analysis, and distribution of rat metallocarboxypeptidase Z. *DNA and cell biology* **1998**, *17*, 311-319, doi:10.1089/dna.1998.17.311.
18. Kalinina, E.; Biswas, R.; Berezniuk, I.; Hermoso, A.; Aviles, F.X.; Fricker, L.D. A novel subfamily of mouse cytosolic carboxypeptidases. *FASEB journal : official publication of the Federation of American Societies for Experimental Biology* **2007**, *21*, 836-850, doi:10.1096/fj.06-7329com.
19. Rodriguez de la Vega Otazo, M.; Lorenzo, J.; Tort, O.; Aviles, F.X.; Bautista, J.M. Functional segregation and emerging role of cilia-related cytosolic carboxypeptidases (CCPs). *FASEB journal : official publication of the Federation of American Societies for Experimental Biology* **2013**, *27*, 424-431, doi:10.1096/fj.12-209080.
20. Tanco, S.; Tort, O.; Demol, H.; Aviles, F.X.; Gevaert, K.; Van Damme, P.; Lorenzo, J. C-terminomics screen for natural substrates of cytosolic carboxypeptidase 1 reveals processing of acidic protein C termini. *Molecular & cellular proteomics : MCP* **2015**, *14*, 177-190, doi:10.1074/mcp.M114.040360.
